# BMI1 fine-tunes gene repression and activation to safeguard undifferentiated spermatogonia fate

**DOI:** 10.3389/fcell.2023.1146849

**Published:** 2023-04-24

**Authors:** Ruiqi Liu, Yonglin Peng, Wenfei Du, Yunqiang Wu, Wen Zhang, Congxia Hu, Min Liu, Xinhua Liu, Ji Wu, Jielin Sun, Xiaodong Zhao

**Affiliations:** ^1^ Key Laboratory of Systems Biomedicine (Ministry of Education), Shanghai Center for Systems Biomedicine, School of Biomedical Engineering, Shanghai Jiao Tong University, Shanghai, China; ^2^ Stem Cell Research and Translation Center, Nanjing Agricultural University, Nanjing, China; ^3^ Department of Integrative Medicine, Obstetrics and Gynecology Hospital, Fudan University, Shanghai, China; ^4^ Department of Biochemistry and Molecular Biology, School of Basic Medical Sciences, Hangzhou Normal University, Hangzhou, China; ^5^ Bio-X Institutes, Shanghai Jiao Tong University, Shanghai, China

**Keywords:** undifferentiated spermatogonia, spermatogenesis, polycomb repressive complex, epigenetic regulation, BMI1

## Abstract

**Introduction:** Spermatogenesis is sustained by the homeostasis of self-renewal and differentiation of undifferentiated spermatogonia throughout life, which is regulated by transcriptional and posttranscriptional mechanisms. B cell-specific Moloney murine leukemia virus integration site 1 (BMI1), one of spermatogonial stem cell markers, is a member of Polycomb repressive complex 1 (PRC1) and important to spermatogenesis. However, the mechanistic underpinnings of how BMI1 regulates spermatogonia fate remain elusive.

**Methods:** We knocked down BMI1 by siRNA to investigate the role of BMI1 in undifferentiated spermatogonia. Differentially expressed genes were identified by RNA-seq and used for KEGG pathway analysis. We performed ChIP-seq analysis in wild type and BMI1 knockdown cells to explore the underlying molecular mechanisms exerted by BMI1. BMI1-associated alterations in repressive histone modifications were detected via Western blotting and ChIP-seq. Furthermore, we performed mass spectrometry and Co-immunoprecipitation assays to investigate BMI1 co-factors. Finally, we demonstrated the genomic regions occupied by both BMI1 and its co-factor.

**Results:** BMI1 is required for undifferentiated spermatogonia maintenance by both repressing and activating target genes. BMI1 preserves PI3K-Akt signaling pathway for spermatogonia proliferation. Decrease of BMI1 affects the deposition of repressive histone modifications H2AK119ub1 and H3K27me3. BMI also positively regulates H3K27ac deposited genes which are associated with proliferation. Moreover, we demonstrate that BMI1 interacts with Sal-like 4 (SALL4), the transcription factor critical for spermatogonia function, to co-regulate gene expression.

**Discussion:** Overall, our study reveals that BMI1 safeguards undifferentiated spermatogonia fate through multi-functional roles in regulating gene expression programs of undifferentiated spermatogonia.

## Introduction

Germ cells are the only cell type which can transmit genetic information across generations and ensure the life cycle of species. Spermatogonial stem cells (SSCs) sustain spermatogenesis during male lifespan which can self-renew and differentiate to spermatozoa, but SSCs are extremely rare and cannot be prospectively isolated by specific antibodies (Tegelenbosch and de Rooij, 1993). Undifferentiated spermatogonia, including A_single_, A_paired_ and A_aligned_ spermatogonia, are active mitotic germ cells, which retain stem cell potential *in vitro* (Kanatsu-Shinohara et al., 2003; Hara et al., 2014). However, the mechanisms of undifferentiated spermatogonia fate decision are not fully understood.

In development cell identity is specified by epigenetic regulation that maintains cell type-specific transcription programs (Orlando, 2003). The epigenetic regulators Polycomb group (PcG) proteins serve as transcriptional repressors, which are essential for stem cell self-renewal, differentiation and development (Laugesen and Helin, 2014). The two most characterized PcG complexes are the Polycomb repressive complex 1 (PRC1) and 2 (PRC2), which catalyze two repressive histone modifications: monoubiquitination of histone H2A at lysine 119 (H2AK119ub1) and methylation of histone H3 at lysine 27 (H3K27me), respectively (Aranda et al., 2015). In mammals, PRC1 is heterogenous and can be further subclassified into two complexes, namely, canonical PRC1 (cPRC1) and non-canonical PRC1 (ncPRC1) (Aranda et al., 2015). Traditionally, PRC1 suppresses gene expression through ring finger protein 1 A/B (Ring1A/B) mediated H2AK119ub1. However, increasing evidence indicates that PRC1 also can activate gene expression in recent years (Gao et al., 2014; Maezawa et al., 2017).

During mammalian spermatogenesis, PRC1 is involved in the establishment of male germline epigenome and timely directs the expression of germline genes (Hasegawa et al., 2015; Maezawa et al., 2017). Due to the heterogenous nature in composition and genomic localization, the role of PRC1 in spermatogenesis has been incompletely understood. Loss of PRC1 leads to decreased stem cell population and severe differentiation defects, causing male infertility (Maezawa et al., 2017; Dai et al., 2018). Among the PRC1’s subunits, B cell-specific Moloney murine leukemia virus integration site 1 (BMI1, also named PCGF4) is of particular importance in male germline development. BMI1 is mainly expressed in undifferentiated spermatogonia, regulates proliferation of undifferentiated spermatogonia and maintains male fertility (Komai et al., 2014; Dai et al., 2018). Recently, BMI1 has been reported to epigenetically repress Wnt10b/β-catenin signaling to promote spermatogonia stem cell maintenance (Yu et al., 2022). BMI1 can assemble cPRC1 and ncPRC1 with distinct biochemical functions in cellular context-dependent manner (Aranda et al., 2015). In addition, highly expressed BMI1 and Inhibitor of differentiation 4 (ID4) are considered as the markers of SSCs (de Rooij, 2017). However, the underlying mechanisms exerted by BMI1 on undifferentiated spermatogonia remain elusive.

In this study, we demonstrate that BMI1 is required for maintenance of undifferentiated spermatogonia. Mechanistically, BMI1 negatively regulates the expression of genes that are repressors of cell proliferation by facilitating H2AK119ub1 and trimethylation of histone H3 at lysine 27 (H3K27me3) modifications. Decrease of BMI1 causes reduction in modification levels of these repressive epigenetic markers. We also observe that BMI1 positively regulates the expression of genes marked with positive epigenomic modification histone H3 acetylated at lysine 27 (H3K27ac). Moreover, BMI1 can interact with Sal-like 4 (SALL4), the transcription factor required for undifferentiated spermatogonia maintenance and differentiation, to co-regulate expression of target genes. Taken together, this study reveals a critical multi-functional role for BMI1 in modulating gene expression programs in undifferentiated spermatogonia.

## Materials and methods

### Animals

ICR mice were housed in individually ventilated cages at 24°C, relative humidity of 50%, 12 h light/dark. Mice were fed a standard chow diet and *ad libitum* access to water. All animal experiments were approved by the Institution Animal Care and Use Committee, Shanghai Jiao Tong University.

### Undifferentiated spermatogonia isolation and culture

As we recently reported (Song et al., 2020), the undifferentiated spermatogonia were isolated from six-day-old mice according to the method described previously (Kanatsu-Shinohara et al., 2003; Kanatsu-Shinohara et al., 2014). Briefly, testes were digested with collagenase and trypsin. Cells were resuspended in IMDM/FBS medium (Kanatsu-Shinohara et al., 2014) and plated onto dishes coated by 0.2% (w/v) gelatin for differential adherence selection. Subsequently, undifferentiated spermatogonia were collected and cultured on mitomycin C-inactivated mouse embryo fibroblasts (MEFs).

Then the expanded undifferentiated spermatogonia were enriched by differential adherence selection to remove MEFs according to the previous reports with minor modifications (Hasegawa et al., 2015; Chan et al., 2017). In short, cells were digested by trypsin and plated on 0.2% (w/v) gelatin-coated dishes. Then the suspended cells were plated again on a gelatin-coated dish. After three rounds of differential adherence selection, the enriched undifferentiated spermatogonia were used for subsequent analysis.

### Undifferentiated spermatogonia transplantation

Undifferentiated spermatogonia transplantation was performed as we described recently (Liu et al., 2022). Briefly, undifferentiated spermatogonia were infected with green fluorescent protein (GFP) expressing lentivirus. The GFP-labelled undifferentiated spermatogonia were digested and filtered through a 70 μm nylon mesh to obtain single-cell suspension. Trypan blue dye was added into the cell suspension before transplantation to monitor the extent of the injections. Following anesthesia and laparotomy, the efferent ducts of the busulfan-treated recipient mice were carefully dissected from the fat tissue surrounding the epididymis and testis for injection. Fifteen μl cell suspension (10^8^ cells/mL) was injected into each testis via the efferent duct. After 2 months, the recipient mice were sacrificed for analysis of SSC localization.

### Transfection of small interfering RNA

Small interfering RNAs (siRNAs) were synthesized by GenePharma (Shanghai, China). siRNA sequences were listed in Supplementary Table S1. siRNA was delivered by Lipofectamine 3,000 (Invitrogen, USA) according to the manufacturer’s protocol.

### Immunofluorescence (IF)

IF on undifferentiated spermatogonia was performed using adhesion microscope slides (Liusheng, China). Cells were fixed with 4% paraformaldehyde for 20 min at room temperature. Cells were washed with PBS prior to permeabilizing in 0.2% Triton X-100 in PBS and blocked in PBS with 10% fetal bovine serum. Then cells were incubated in primary antibodies followed by the second antibodies. Cell nuclei were stained with DAPI (Sigma, USA). The information of antibodies was listed in Supplementary Table S2.

### RNA extraction and quantitative real time PCR (qPCR)

Total RNA was extracted using TRIzol (Invitrogen, USA). RNA was reverse-transcribed to cDNA by PrimeScript^TM^RT reagent Kit with gDNA Eraser (TAKARA, Japan). Reactions were performed using TB Green Premix EX Taq II (TAKARA, Japan) in ABI StepOne plus real-time PCR system. Primers used in qPCR were listed in Supplementary Table S1. Expression was normalized to *GAPDH*. Three biological replicates were performed.

### Western blotting

Cells were collected after differential adherence selection, then lysed by RIPA lysis buffer (Thermo fisher, USA) with protease inhibitor cocktail (Roche, Switzerland). The concentration of sample was measured by BCA Protein Assay Kit (Pierce, USA). Protein lysates were separated in sodium dodecyl sulfate polyacrylamide gel electrophoresis system (SDS-PAGE) and transferred to PVDF membranes (Millipore, USA). Membranes were blocked in 5% BSA/TBST for 2 h at room temperature. Subsequently, membranes were incubated in the primary antibodies overnight at 4°C. Then membranes were washed twice by TBST followed by incubation with HRP-conjugated secondary antibodies for 2 h at room temperature. Finally, membranes were washed twice by TBST. The results were visualized using ECL start Western Blotting Substrate (GE Healthcare Life Sciences, USA) and analyzed by ImageJ (National Institutes of Health, USA). The information of antibodies was listed in Supplementary Table S2.

### Cell counting kit-8 (CCK-8) assay

Cells were cultured in 96-well plates and transfected with siRNA. For each time point, 10 μL CCK-8 solution (Sangon Biotech, China) was added to culture medium and incubated for 1 h in the incubator. The absorbance was measured at 450 nm wavelength. Three biological replicates were performed.

### Cell cycle analysis

Cells were collected and washed by cold PBS, then fixed with 70% cold ethanol at 4°C overnight. The fixed cells were washed with PBS twice and resuspended in 500 μL propidium iodide (PI) solution (50 μg/mL, Thermo Fisher, USA) with RNase A (50 μg/mL, Thermo Fisher, USA) at 37°C 30 min. Data were detected at 488 nm using BD LSRFortessa (BD Biosciences, USA). Three biological replicates were performed.

### TUNEL staining

Undifferentiated spermatogonia were placed on microscope slides (Liusheng, China) and stained with Fluorescein (FITC) Tunel Cell Apoptosis Detection Kit (Servicebio, China) according to the manufacturer’s instructions. Cell nuclei were stained with DAPI (Sigma, USA). Three biological replicates were performed.

### RNA-seq library preparation and analysis

Total RNA was isolated by Trizol (Invitrogen) according to the manufacturer’s protocol. mRNA was purified by NEBNext Poly(A) mRNA Magnetic Isolation Beads (NEB, USA). The RNA libraries of two biological replicates were constructed using the NEBNext Ultra Directional RNA Library PrepKit (NEB, USA). All libraries were sequenced at 150 bp paired-end on HiSeq 2000 (Illumina, USA).

Raw sequencing reads were subjected to Trim-Galore (Version 0.6.5, https://github.com/FelixKrueger/TrimGalore) with parameter “--paired--stringency 3 -q 20 --length 20.” Then, the qualified paired-end reads were mapped to the mouse genome (mm10) using Hisat2 (version 2.2.1) (Kim et al., 2019). Gene expression levels were calculated by featureCounts (version 2.0.1) (Liao et al., 2014) and transformed into fragments per kilobase of exon per million fragments mapped (FPKM). Differentially expressed gene (DEG) analysis was according to the method in the recent report (Yao et al., 2018). Briefly, DEGs were calculated log_2_ fold change value for each gene in paired *Bmi1* knockdown and control samples. Upregulated and downregulated genes were defined as 1.5-fold change cutoff, and only genes with a mean FPKM value >1 in at least one condition were included. To visualize RNA-seq signal at individual genomic regions, we used the Integrative Genomics Viewer (IGV) (Thorvaldsdóttir et al., 2013).

### Chromatin immunoprecipitation (ChIP) and analysis

ChIP was performed as described previously (Maezawa et al., 2018). Briefly, 4 × 10^6^ cells were used for each ChIP-seq experiment. Cells were cross-linked using a final concentration of 1% formaldehyde (Thermo Fisher, USA) at room temperature for 10 min, then stopped by a final concentration of 125 mM Glycine (Sangon, China) at room temperature for 5 min, subsequently washed twice with PBS. Nuclei were isolated according to the study previously described (Maezawa et al., 2018). Chromatin was sheared by Covaris sonicator (Cavaris, USA). The fragmented chromatin was immunoprecipitated with Magna ChIP™ Protein A + G Magnetic beads (Millipore, USA) coupled with antibodies overnight at 4°C. Then, the beads were washed with low salt, high salt, LiCl and TE buffer. Bound chromatin was eluted and reverse crosslinked. ChIP and input libraries of two biological replicates were generated using KAPA Hyper Prep Kit (KAPA, USA) according to the manufacturer’s instruction. Libraries were sequenced at 150 bp paired-end on HiSeq 2,000 (Illumina, USA). The information of antibodies was listed in Supplementary Table S2.

The adapter and low-quality sequences were trimmed from 3′ to 5′ ends by Trim-Galore (Version 0.6.5, https://github.com/FelixKrueger/TrimGalore). Subsequently, the preprocessed reads were aligned to the mouse reference genome (mm10) using Bowtie2 (version 2.4.4) (Langmead and Salzberg, 2012). Then, the aligned reads were converted to bam format using SAMtools (Li et al., 2009) and duplicates were removed by Picard software (version 2.27.3) (https://github.com/broadinstitute/picard). We combined bam files of two replicates into one file to identify peaks as we did previously (Zhang et al., 2016). And then peaks of ChIP-seq were called using MACS2 software (Zhang et al., 2008) (*p*-value <10^−5^) with the parameters of “-f BAMPE -g mm -p 1e-5” and “-f BAMPE -g mm--broad -p 1e-5 --broad-cutoff 0.01” for calling sharp peak and broad peak, respectively. Peaks were annotated by annotatePeaks.pl function in the homer package (version 4.11) (Heinz et al., 2010). We identified target genes and the distance of every peak to the nearest TSS. Peaks were filtered in depending on their localization as −2.5 kb from TSS to TES and converted into official gene symbols. The density of histone signal was calculated by deepTools (Version 3.5.1) (Ramírez et al., 2014). To visualize ChIP-seq signal at individual genomic regions, we used the IGV (Thorvaldsdóttir et al., 2013). For H2AK119ub1 and H3K27me3, differential peaks in two conditions of wild type (WT) and knockdown (KD) were detected by bdgdiff command in MACS2 software (Zhang et al., 2008).

### ChIP-qPCR

ChIP-qPCR was performed as we described previously (Song et al., 2020). The primer sequences were listed in Supplementary Table S1. Reactions were carried out with TB Green Premix EX Taq II (TAKARA, Japan) in accordance with the manufacturer’s instructions. The qPCR experiments were performed in triplicate and the results were normalized to the input DNA and analyzed with the ΔΔCT method.

### Co-immunoprecipitation (Co-IP) and mass spectrometry

Co-IP was performed as the cold spring harbor protocol described with minor modifications (DeCaprio and Kohl, 2017). Cells were lysed by M-PER™ Mammalian Protein Extraction Regent (Thermo Fisher, USA) with protease inhibitor cocktail (Roche, Switzerland) on ice 45 min. Lysates were centrifugated at 13,000 g for 20 min at 4°C. The supernatant was collected and quantified by BCA Protein Assay Kit (Pierce, USA). Protein was equally divided to each IP, and incubated with primary antibodies at 4°C overnight and then mixed with Protein A/G Plus-Agarose (Santa Cruz Biotechnology, USA) for 2 h at 4°C. The information of antibodies was listed in Supplementary Table S2. The beads were washed three times with IP buffer. The bound proteins were eluted by SDS-buffer at 100°C for 10 min, then loaded onto SDS-PAGE gels for sliver staining (Sangon, China) according to manufacturer’s instructions or Western blotting. Two biological replicates were performed.

Protein bands from IgG and BMI1 antibody captured were identified using mass spectrometry by Instrumental Analysis Center of Shanghai Jiao Tong University. The mass spectrometry assay was performed as the previous study described (Jiang et al., 2018). Briefly, the gels were digested with trypsin. The resulting peptides were analyzed using an LC system (Nano Pump, Ultimate 3,000, Dionex, Thermo Fisher, USA) coupled with an ESI-Q-TOF mass spectrometer (MaXis, Impact, Bruker Daltonik, Germany). The MS/MS spectra data were searched against the NCBInr database for protein identification using the Mascot (Matrix Science, MA) suite with a precursor ion mass tolerance of 20 ppm and fragment ion mass tolerance of 0.05 Da. The proteins identified by mass spectrometry with a fold-change of ≥2 and a minimum of two identified peptides were considered as potential BMI1 interactors.

### Gene Ontology (GO) and Kyoto Encyclopedia of Genes and Genomes (KEGG) analysis

Gene Ontology (GO) enrichment annotation and Kyoto Encyclopedia of Genes and Genomes (KEGG) analysis were performed by DAVID (Huang da et al., 2009), the terms with *p*-value less than 0.05 were considered significant.

### Statistical analysis

The differences were analyzed by Student’s *t*-test. Data were showed as means ± s.d. Statistical significance was defined as *p* < 0.05. ***p < 0.001. **p < 0.005. *p < 0.05.

## Results

### BMI1 is indispensable for the undifferentiated spermatogonia maintenance

To investigate the role of BMI1 in undifferentiated spermatogonia, we isolated cells from testis of six-day-old ICR mouse. The enriched cells formed grape-like clusters upon MEFs (Supplementary Figures S1A, B). To verify the identity of these cells, the expression of marker genes in undifferentiated spermatogonia was detected at protein and mRNA levels, respectively. The protein expression of ID4, PLZF and MVH was confirmed by immunofluorescence (Supplementary Figures S1C–E). Compared with those from testis, the mRNA levels of *Plzf*, *Gfrα1* and *Id4* were significant higher in the enriched cells (Supplementary Figure S1F). Finally, GFP labelled enriched cells migrated into and localized in the niche of busulfan-treated recipient after transplantation, indicating the capacity of homing and reconstitution of spermatogenesis (Supplementary Figure S1G). These results indicate that the enriched cells are undifferentiated spermatogonia.

**FIGURE 1:**
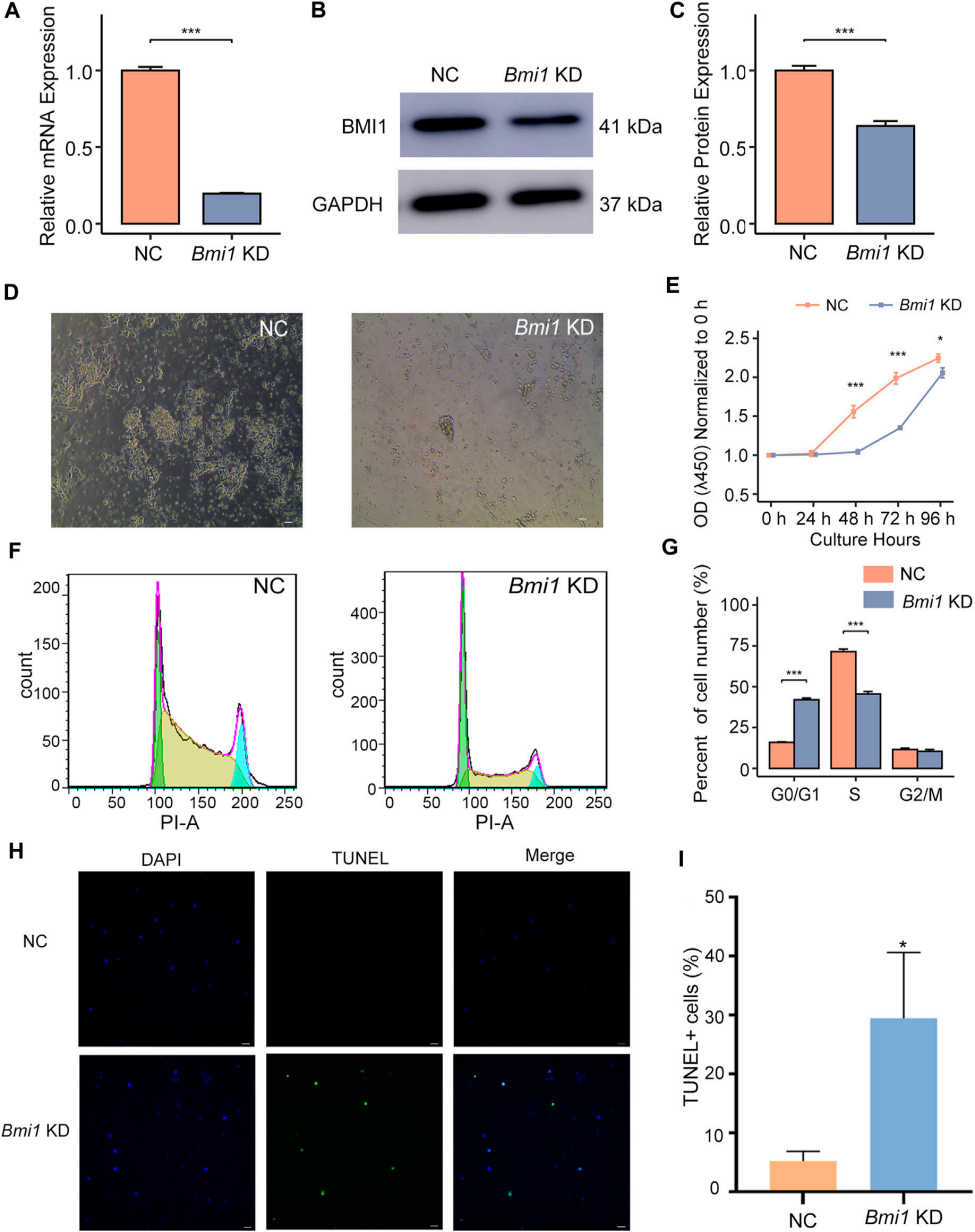
*Bmi1* knockdown disturbs undifferentiated spermatogonia maintenance. **(A)** qPCR analysis of *Bmi1* mRNA expression in control (NC) and *Bmi1* knockdown cells (KD) 2 days after transfection. Data are showed as means ± s.d. and derived from three independent experiments. **(B)** Western blotting analysis of BMI1 expression in NC and *Bmi1* KD cells 2 days after transfection. **(C)** The protein expression levels of BMI1 are measured by Image J analysis. **(D)** Representative images of NC and *Bmi1* KD cells 2 days after transfection. Scale bar, 20 μm. **(E)** CCK-8 assay of undifferentiated spermatogonia treated with *Bmi1* siRNA or NC siRNA for 4 days. **(F)** Cell cycle distribution is determined by flow cytometry. **(G)** The proportion of cell number in G0/G1, S and G2/M state is determined by flow cytometric analysis. **(H)** TUNEL staining of NC and *Bmi1* KD cells 2 days after transfection. Scale bar, 20 μm. **(I)** Quantification of TUNEL^+^ cells. Data are showed as means ± s.d. and derived from three independent experiments.

To further understand the role of BMI1, we knocked down *Bmi1* by short interfering RNA (siRNA). *Bmi1* knockdown (KD) was verified by qPCR and Western blotting (Figures 1A–C). After 48 h culture, we found that the colonies of *Bmi1* KD cells were smaller and colony number was less than those of control group (Figure 1D), indicating the self-renewal is compromised by *Bmi1* knockdown. Meanwhile, reduction of *Bmi1* suppressed spermatogonia proliferation in day 2 of culture (Figure 1E). Through analysis of cell cycle phase using flow cytometry, we noticed the significant increase in the percentage of cell number in the G0/G1 phase, and the decrease in the percentage of cell number in the S phase in *Bmi1* KD cells relative to control cells (Figures 1F, G). In addition, we found that decrease of *Bmi1* induced cell apoptosis (Figures 1H, I). Collectively, these findings indicate that BMI1 is required for the undifferentiated spermatogonia maintenance.

### BMI1 regulates PI3K-Akt signaling pathway to maintain the undifferentiated spermatogonia proliferation

**FIGURE 2:**
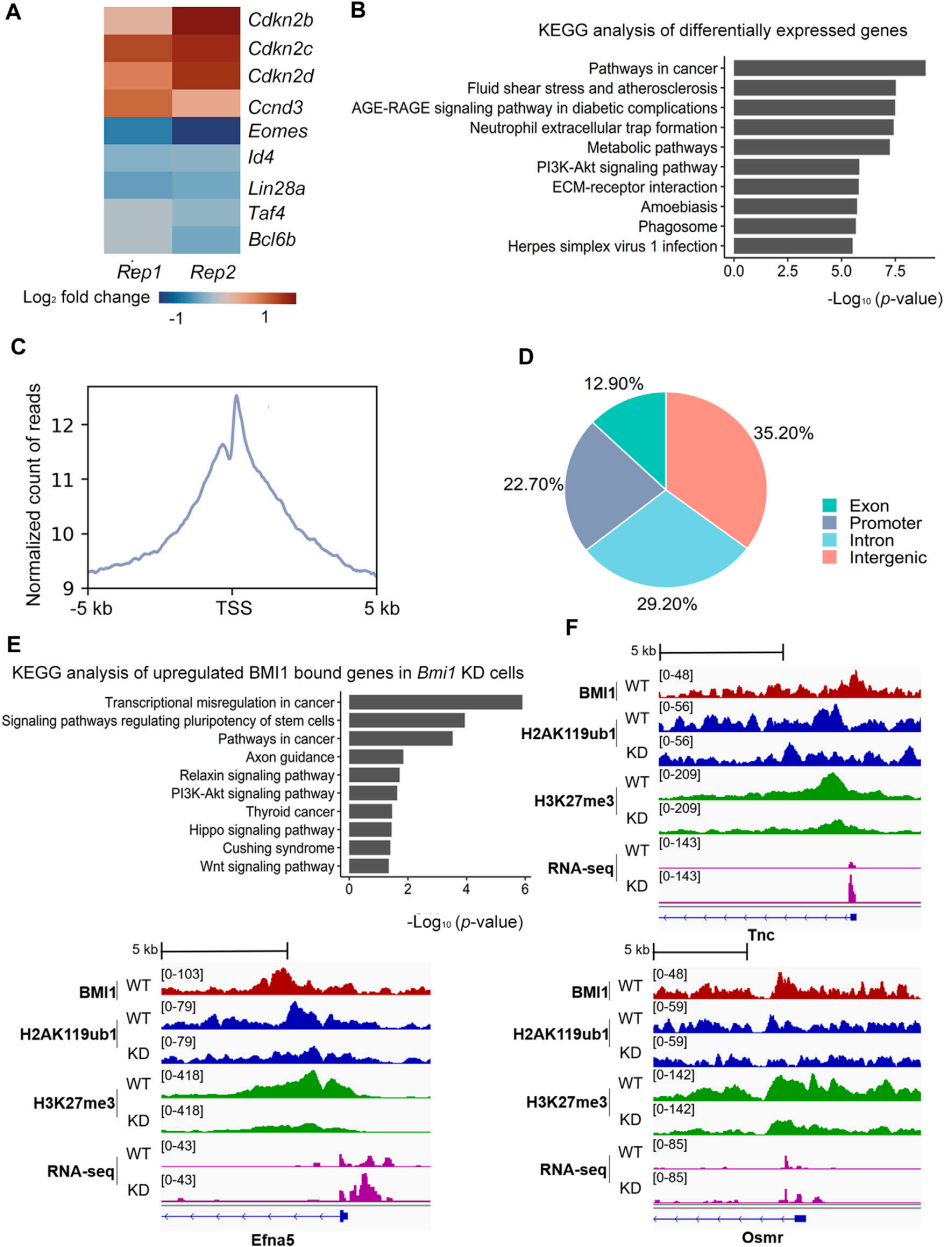
BMI1 regulates PI3K-Akt signaling pathway to maintain undifferentiated spermatogonia fate. **(A)** Heatmap illustrating the expression changes of selected genes related with proliferation and stemness. Results are showed as log_2_ fold change in wild type and knockdown cells. Each lane corresponds to an independent paired biological sample. **(B)** KEGG analysis of differentially expressed genes. Results are expressed as -log_10_ (*p*-value). **(C)** TSS (±5 kb) enrichment plot of BMI1 ChIP-seq at target sites. **(D)** Genome-wide distribution of BMI1 binding regions. **(E)** KEGG analysis of upregulated BMI1 bound genes in *Bmi1* knockdown cells. Results are expressed as -log_10_ (*p*-value). **(F)** IGV shows the binding peaks of BMI1, H2AK119ub1 and H3K27me3 ChIP-seq results together with RNA-seq data from WT and *Bmi1* KD cells at *Tnc*, *Efna5* and *Osmr* loci.

Having observed the requirement of BMI1 in the proliferation of undifferentiated spermatogonia, we then sought to understand the underlying mechanisms of BMI1 biological function. Thus, we performed RNA-seq of two biological replicates in both wild type and *Bmi1* knockdown spermatogonia populations. The replicates are fairly correlated (Supplementary Figure S2A). Compared with wild type spermatogonia, 4,318 genes are differentially expressed in *Bmi1* knockdown population, including 2,338 upregulated and 1,980 downregulated genes (Supplementary Table S3). Among the downregulated genes we found the stemness-related transcription factors, such as *Bcl6b*, *Lin28a* and *Id4* (Figure 2A). Meanwhile, the expression levels of *Cdkn2b*, *Cdkn2c* and *Cdkn2d* that negatively regulate cell proliferation are increased (Figure 2A). GO analysis indicates that the terms of spermatogenesis and cell differentiation are enriched in downregulated genes and the terms of negative regulated cell proliferation and positive regulation of apoptotic process are enriched in the upregulated genes (Supplementary Figures S3A, B).

To further explore the underlying molecular mechanisms of BMI1-mediated proliferation of undifferentiated spermatogonia, we performed KEGG pathway analysis on differentially expressed genes. The results show that BMI1-dependent genes are highly enriched for gene networks regulating PI3K-Akt signaling pathway, which is critical for spermatogonia self-renewal (Figure 2B). We next interrogated the genome-wide distribution of BMI1 with chromatin immunoprecipitation followed by massive parallel sequencing (ChIP-seq) (Supplementary Figure S2B). BMI1 ChIP-seq yielded 2,645 peaks corresponding to 1,135 genes (Supplementary Table S4). We observed a preferential distribution of BMI1 around transcription start sites (TSS) of genes (Figure 2C). About 22.7% of BMI1 binding regions are near promoter regions (from −2.5 kb to 0.5 kb around TSS), 35.2% of the BMI1-binding regions are located in the intergenic regions, a significant number of BMI1 binding regions fall within genes, with 29.2% in the introns and 12.9% in the exons (Figure 2D).

Combination analysis of BMI1 ChIP-seq and RNA-seq data indicated that 146 BMI1-bound genes are upregulated, while 113 BMI1-bound genes are downregulated after *Bmi1* knocked down. These upregulated genes are enriched in PI3K-Akt signaling pathway (Figure 2E), further suggesting that BMI1 might direct PI3K-Akt signaling pathway to maintain undifferentiated spermatogonia. Included within these upregulated genes are *Tnc*, *Efna5* and *Osmr*, the negative regulators of PI3K-Akt signaling pathway. In particular, three genes are repressed by BMI1 in wild type by H3K27me3 and H2AK119ub1, because *Bmi1* knockdown leads to significant decrease in modification levels of H2AK119ub1 and H3K27me3 at these genes and higher expression (Figure 2F). Taken together, these data suggest that BMI1 regulates PI3K-Akt activity to maintain the undifferentiated spermatogonia proliferation.

### BMI1 assembles canonical PRC1 in undifferentiated spermatogonia

**FIGURE 3:**
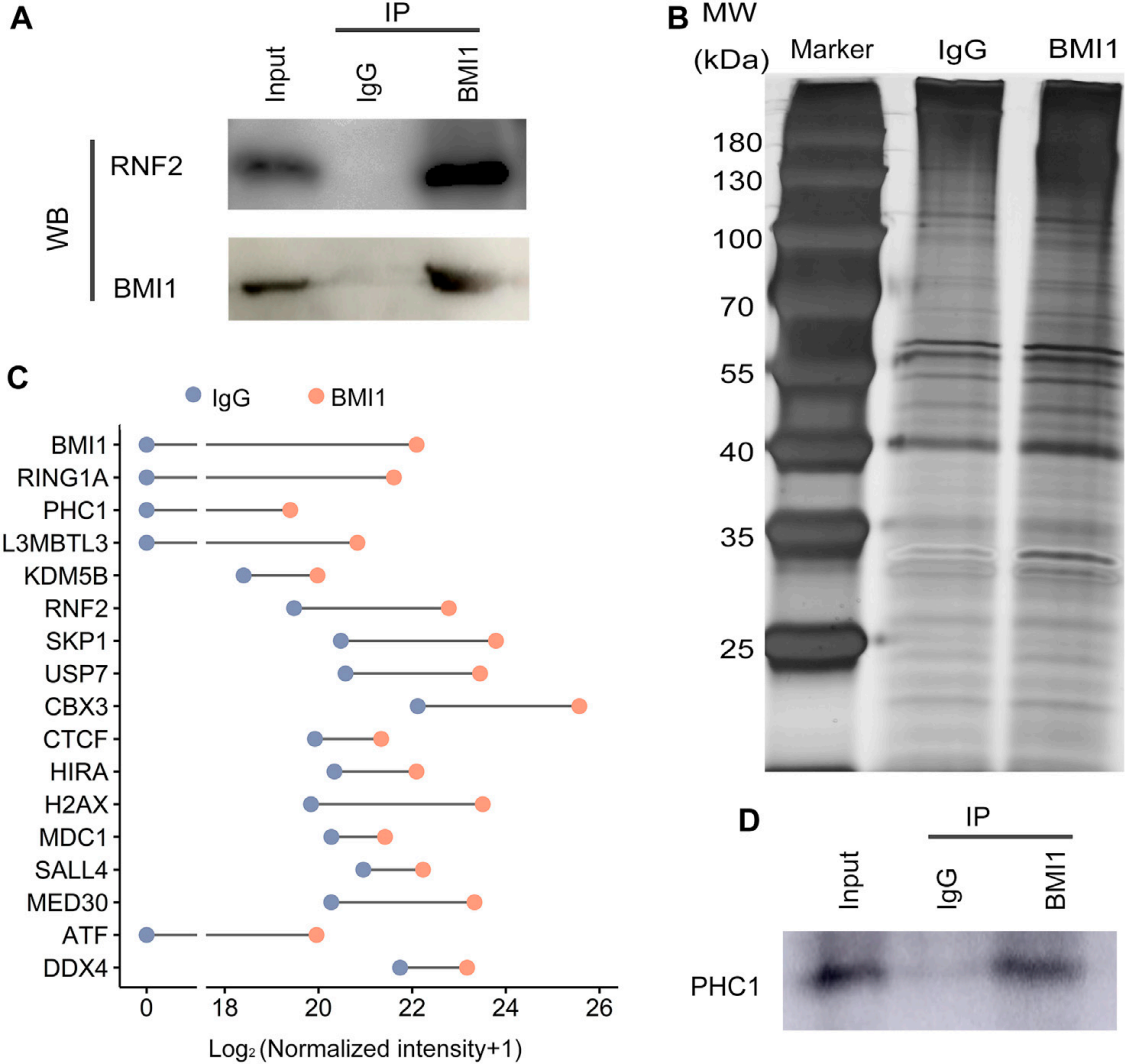
BMI1 interactomes. **(A)** Detection the interaction of BMI1 and RNF2 in undifferentiated spermatogonia by Co-IP. **(B)** Silver-stained SDS-PAGE gel from IgG and BMI1 purifications. **(C)** BMI1-associated proteins are identified by mass spectrometry. **(D)** Confirmation of the interaction of BMI1 and PHC1.

Mammalian PRC1 complexes are quite heterogeneous. Different combinations of the subunits give rise to functionally distinct PRC1 complexes, which could be broadly classified into cPRC1 and ncPRC1. As BMI1 is the key component of PRC1 complex, we then asked whether BMI1 assembles cPRC1 or ncPRC1 in undifferentiated spermatogonia. Given the observation that BMI1 also exerts its function independently of PRC1, we firstly asked whether BMI1 is involved the assembly of PRC1, and examined the interaction of BMI1 with RNF2 (the core member of PRC1) through Co-IP assay. We found BMI1 physically associates with RNF2 (Figure 3A), suggesting that BMI1 assembles PRC1 in undifferentiated spermatogonia. To further investigate the yet-unknown interactome of BMI1, we purified protein extracts for immunoprecipitation with anti-BMI1 antibody (Figure 3B), and the immunoprecipitants were analyzed by quantitative mass spectrometry. Consistent with the RNF2 Co-IP result, we identified the core members of the PRC1 complex, such as RING1A and RNF2 (Figure 3C). In particular, we observed the association of BMI1 with PHC1, a subunit exclusively present in cPRC1 (Figure 3C). We however did not detect the association of BMI1 with ncPRC1 specific subunits RYBP and YAF2. These observations suggest that BMI1-assembled cPRC1 is dominant in the undifferentiated spermatogonia. In addition to the subunits of PRC1, we demonstrated that BMI1 is associated with some transcription factors and germ cell-specific markers (Figure 3C; Supplementary Table S5). The interaction of BMI1 with PHC1 was subsequently confirmed by immunoprecipitation combined with Western blotting (Figure 3D).

### BMI1 affects the distribution of H2AK119ub1 and H3K27me3

**FIGURE 4:**
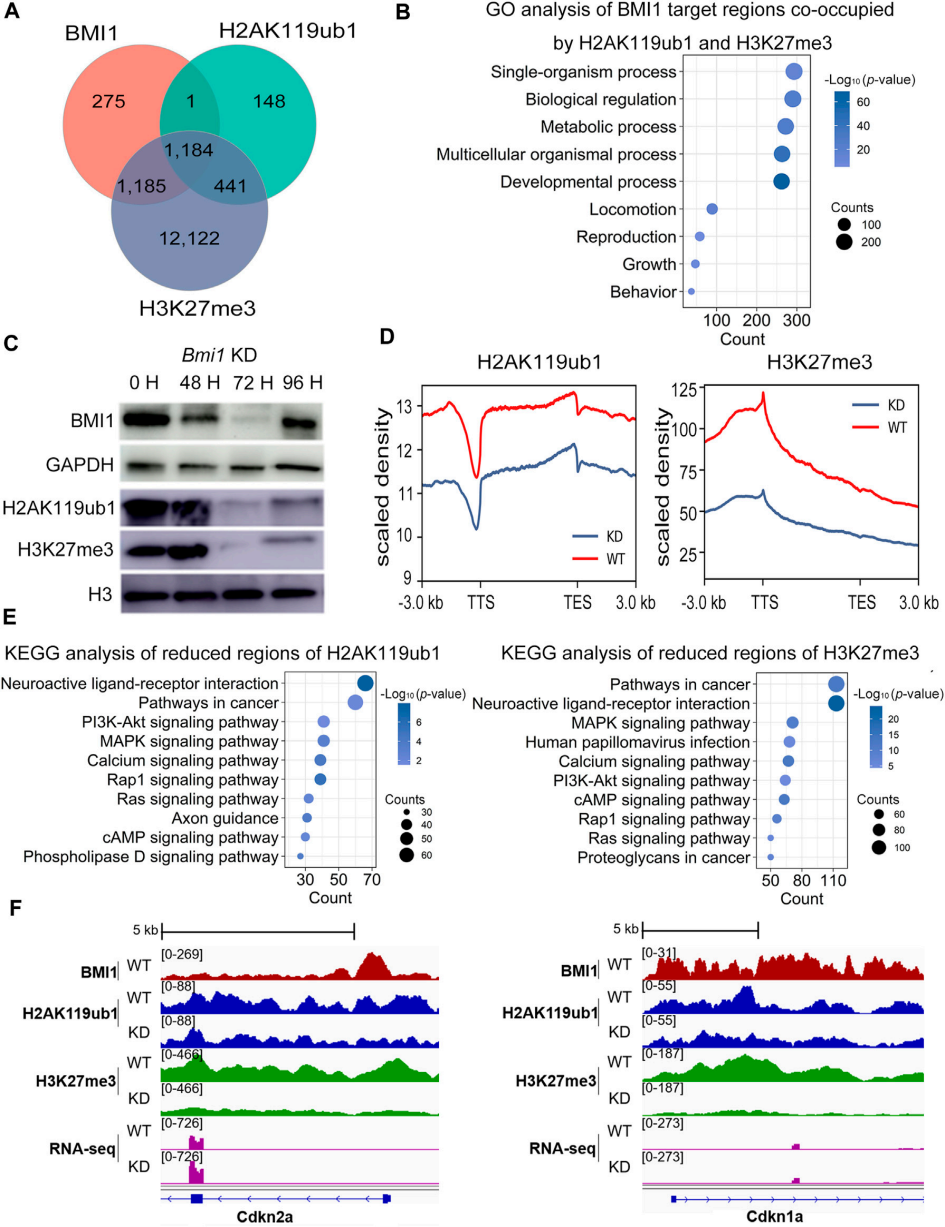
*Bmi1* knockdown impairs repressive chromatin and disturbs undifferentiated spermatogonia state. **(A)** Venn diagram shows the overlap among target regions of BMI1, H2AK119ub1 and H3K27me3. **(B)** GO analysis of biological functions of BMI1 target regions co-occupied by H2AK119ub1 and H3K27me3. **(C)** Western blotting analysis of H2AK119ub1 and H3K27me3 levels during *Bmi1* knockdown. **(D)** Density profile of H2AK119ub1 and H3K27me3 signal tracks, after *Bmi1* knockdown. **(E)** KEGG analysis of reduced regions of H3K27me3 and H2AK119ub1 after *Bmi1* knockdown. **(F)** IGV shows the binding profiles of BMI1, H2AK119ub1 and H3K27me3 ChIP-seq results together with RNA-seq data from WT and *Bmi1* KD cells at genes of *Cdkn2a* and *Cdkn1a.*

cPRC1 drives gene repression through RING1-catalyzed H2AK119ub1 and is recruited in a sequential manner to genomic regions marked by pre-existing H3K27me3, while BMI1 promotes RING1 catalytic activity. We next analyzed the genome-wide distribution of PRC1-mediated H2AK119ub1 and PRC2-mediated H3K27me3. The enriched undifferentiated spermatogonial cells were subjected to ChIP-seq analysis (Supplementary Figure S2B). Comparison of ChIP-seq peaks between BMI1 and histone modifications revealed that near 90% of BMI1 binding regions are marked by H3K27me3, whereas 44.8% of BMI1 bound regions are marked by H2AK119ub1 (Figure 4A; Supplementary Table S6). This observation is consistent with the proposed action model of Polycomb proteins in which PRC1 is recruited in a hierarchical manner to the H3K27me3 modified genomic regions. Moreover, BMI1 target profile is similar with H2AK119ub1 and H3K27me3 profiles (Supplementary Figure S4A). GO analysis indicates that BMI1-bound genes with H2AK119ub1 and H3K27me3 modifications are involved in developmental process, multicellular organismal process and reproduction (Figure 4B).

To further investigate the effect of BMI1 on the genome-wide distribution of H2AK119ub1 and H3K27me3, we carried out ChIP-seq analysis using H2AK119ub1 and H3K27me3 antibodies in both wild type and *Bmi1* knockdown undifferentiated spermatogonial cells (Supplementary Figures S2B, C). Western blotting showed that both H2AK119ub1 and H3K27me3 levels are decreased upon *Bmi1* knockdown (Figure 4C). Compared with the wild type spermatogonial cells, the global ChIP-seq signals of H2AK119ub1 and H3K27me3 are decreased in the *Bmi1* knockdown counterpart (Figure 4D). These observations suggest that BMI1 is required to maintain these two repressive histone modifications in undifferentiated spermatogonia. Gene functional enrichment analysis reveals that the terms of PI3K-Akt signaling, cell differentiation and cell development are enriched in the genomic regions with decreased levels of H2AK119ub1 and H3K27me3 (Figure 4E, Supplementary Figures S4B, C). In particular, we observed *Bmi1* knockdown triggers the reduction of H2AK119ub1 and H3K27me3 levels at the repressors of PI3K-Akt signaling pathway (Figure 2F) and the cell-cycle inhibitors *Cdkn2a* and *Cdkn1a* (Figure 4F); accordingly, the transcription levels of these genes are increased (Figures 2F, 4F). Overall, these data suggest that BMI1 represses the negative regulators of PI3K-Akt pathway by deposition of H2AK119ub1 and H3K27me3.

### BMI1 positively regulates the expression of genes for cell proliferation

**FIGURE 5:**
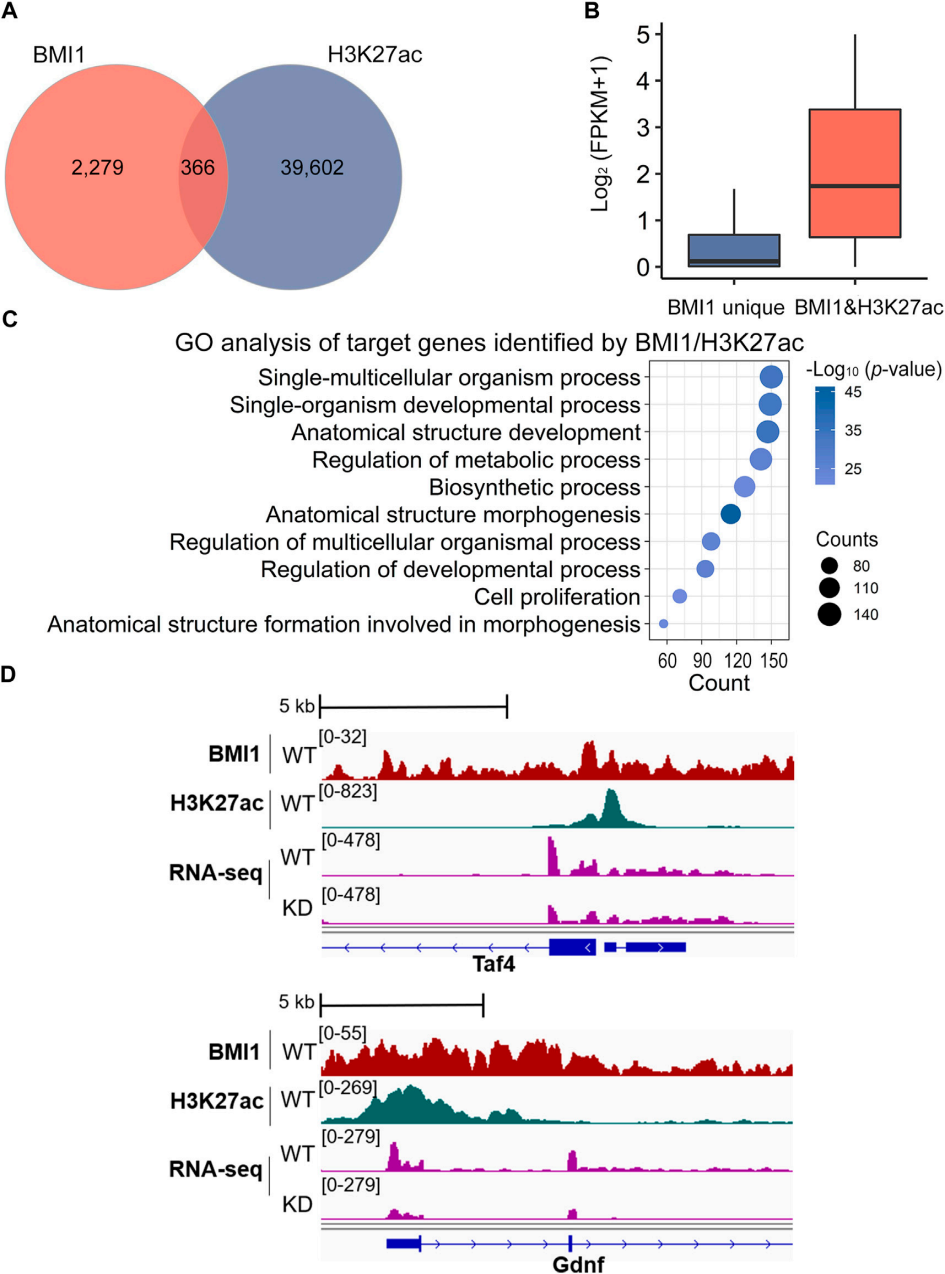
BMI1 maintains cell proliferation by H3K27ac modification. **(A)** Venn diagram shows the overlap among target regions of BMI1 and H3K27ac. **(B)** Expression analysis of genes targeted by BMI1 unique and BMI1/H3K27ac. **(C)** GO analysis of target genes identified by BMI1/H3K27ac. **(D)** IGV browser views for BMI1 and H3K27ac at self-renewal and proliferation genes.

As a subunit of PRC1 that deposits repressive histone modification H2AK119ub1, BMI1 is frequently associated with gene repression. However, when we revisited our RNA-seq data, we identified 113 BMI1-bound genes downregulated in *Bmi1* knockdown cells. We thus asked whether BMI1 positively regulates gene expression. To address this question, we performed ChIP-seq analysis of H3K27ac of two biological replicates (Supplementary Figure S2B), a histone modification mark associated with active transcription. Analysis of ChIP-seq peaks for BMI1 revealed that 13.8% of BMI1 binding regions (*n* = 366) contain peaks for H3K27ac (Figure 5A; Supplementary Table S6). We next assessed the expression status of BMI1 target genes with the presence or absence of H3K27ac, and observed that the expression of BMI1 target genes with H3K27ac is significantly higher than those without H3K27ac (Figure 5B). These data suggest that BMI1 may activate a considerable number of genes in undifferentiated spermatogonia. GO analysis reveals the term of cell proliferation is enriched in H3K27ac marked BMI1 target genes (Figure 5C). Importantly, among these activated genes, we observed several self-renewal and proliferation-related genes including *Taf4* and *Gdnf*, and their expression levels are decreased in *Bmi1* knockdown cells (Figure 5D). The results suggest a link between BMI1 and these targets in regulation of undifferentiated spermatogonia.

### BMI1 interacts with SALL4 to repress the differentiation-related genes in undifferentiated spermatogonia

**FIGURE 6:**
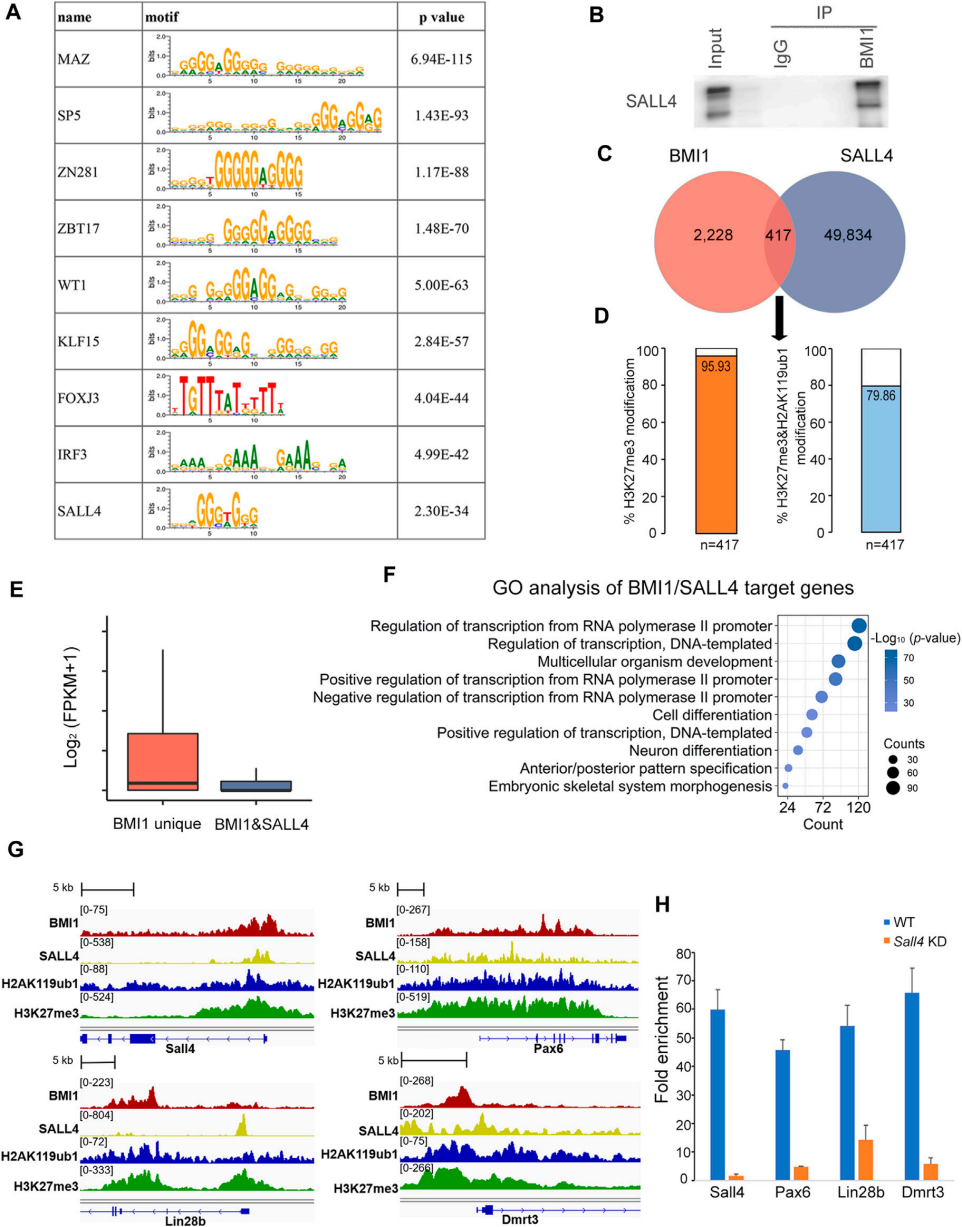
BMI1 interacts with SALL4 to work together for gene repression. **(A)** Motif analysis at BMI1 target regions. **(B)** Confirmation of the interaction of BMI1 and SALL4. **(C)** Venn diagram shows the overlap among target regions of BMI1 and SALL4. **(D)** Percentage of BMI1 and SALL4 co-bound genomic regions modified by H3K27me3 and H2AK119ub1. **(E)** Box plot represents the gene expression values in BMI1 alone target genes and BMI1/SALL4 target genes. **(F)** GO analysis of BMI1/SALL4 target genes. **(G)** IGV browser views for BMI1 and SALL4 co-targeted genes. **(H)** ChIP-qPCR shows the changes in BMI1 binding at the selected genomic regions in *Sall4* KD cells. Data are showed as means ± s.d. and derived from three independent experiments.

Unlike PcG response elements (PREs) that direct PRC binding in *Drosophila*, there are quite few PREs reported in mammals. A proposed action model is that PcG proteins are recruited to the specific loci of mammalian genome by interaction with sequence-specific DNA binding proteins. Prompted by this model, we utilized MEME to analyze the binding motifs among BMI1 ChIP-seq peaks. Among the top 10 enriched consensus-binding sites we found the motif of SALL4, the critical transcription factor for undifferentiated spermatogonia maintenance and differentiation (Figure 6A). Intriguingly, we observed that SALL4 is a member of BMI1 interactome in our mass spectrometry analysis (Figure 3C). We further confirmed the interaction between SALL4 and BMI1 using Co-IP assay (Figure 6B). Analysis of ChIP-seq peaks for BMI1 revealed that 15.8% of BMI1 binding regions (*n* = 417) contain peaks for SALL4 (Figure 6C; Supplementary Table S6). We further observed that 95.93% of BMI1 and SALL4 co-bound genomic regions are modified by H3K27me3 (*n* = 400), whereas 79.86% of BMI1 and SALL4 co-bound genomic regions are deposited by H2AK119ub1 and H3K27me3 (*n* = 333) (Figure 6D). We then found that the genes occupied by both BMI1 and SALL4 are expressed lower than those occupied by BMI1 alone (Figure 6E). GO analysis indicates that these genes co-occupied by BMI1 and SALL4 are involved in cell differentiation, such as *Sall4*, *Pax6*, *Lin28b* and *Dmrt3* (Figures 6F, G). To further understand the influence of BMI1-SALL4 interaction on the target genes, we performed BMI1 ChIP-qPCR in both WT and *Sall4* KD cells, and observed the significant reduction in BMI1 binding at the examined genomic regions in *Sall4* KD cells (Figure 6H). Collectively, these data suggest that BMI1 is recruited by SALL4 to repress the differentiation-related genes in undifferentiated spermatogonia.

## Discussion

During distinct stages of male germ cell development BMI1 is expressed predominantly by undifferentiated spermatogonia and required for their maintenance (Komai et al., 2014; Dai et al., 2018). *Bmi1* is a member of *Pcgf* gene family that represses gene expression by enhancing ubiquitination of histone H2A (Cao et al., 2005) and renders developmental context-specific activity to PRC1. Yet, relatively little is known about how BMI1 exerts its regulatory influence in undifferentiated spermatogonia.

In this study, we elucidated the molecular mechanism of BMI1 as an epigenetic regulator to control undifferentiated spermatogonia fate. Consistent with previous findings (Komai et al., 2014; Dai et al., 2018), we observed that *Bmi1* knockdown reduces the self-renewal activity of undifferentiated spermatogonia, induces cell cycle arrest and cell apoptosis. BMI1 modulates expression of more than four thousand genes. Among the downregulated genes, several members of PI3K-Akt signaling pathway raised our attention. PI3K-Akt pathway is critical for the self-renewal of undifferentiated spermatogonia (Lee et al., 2007). We found its negative regulators *Tnc* (Tong et al., 2020), *Efna5* (Ricci et al., 2020) and *Osmr* (Tian et al., 2019) are bound by BMI1 and modified by H2AK119ub1 and H3K27me3. These findings suggest BMI1 contributes to the activation of PI3K-Akt pathway by facilitating the deposition of repressive histone modifications on its negative regulators. A recent study reported that BMI1 represses Wnt10b/β-catenin signaling for undifferentiated spermatogonia maintenance (Yu et al., 2022). In our study, we found that BMI1 binds Wnt10b modified with H2AK119ub1 and H3K27me3.

In addition to the well-recognized repressive effect on gene expression, a couple of reports revealed that PRC1 complex can be involved in gene activation (Gao et al., 2014; Maezawa et al., 2017). Here, we also observed BMI1 upregulates a considerable number of genes. The positively regulated genes *Taf4* and *Gdnf* (Song and Wilkinson, 2014) are deposited by active histone modification H3K27ac and have been reported to be actively involved in undifferentiated spermatogonia. Our findings indicate BMI1 fine-tunes gene repression and activation to safeguard the maintenance of undifferentiated spermatogonia.

The transcription factor SALL4 is important for fate decision of postnatal spermatogonial progenitor cells (Hobbs et al., 2012). Although the binding targets of SALL4 have been deciphered in undifferentiated spermatogonia genome (Lovelace et al., 2016), SALL4 modulates gene expression by associating with NuRD or RNF2, and plays distinct roles in spermatogonial population (Chan et al., 2017; Maezawa et al., 2017). In addition to these reported co-factors, in this study we found SALL4 interacts with BMI1, and selectively deposits the repressive histone modifications H2AK119ub1 and H3K27m3 on differentiation-related genes, such as *Sall4* (Hobbs et al., 2012), *Pax6* (Kimura et al., 2015), *Lin28b* (Gaytan et al., 2013) and *Dmrt3* (Yamaguchi et al., 2006). These observations suggest SALL4 interacts with diverse chromatin modifiers to maintain the undifferentiated spermatogonial activity.

**FIGURE 7:**
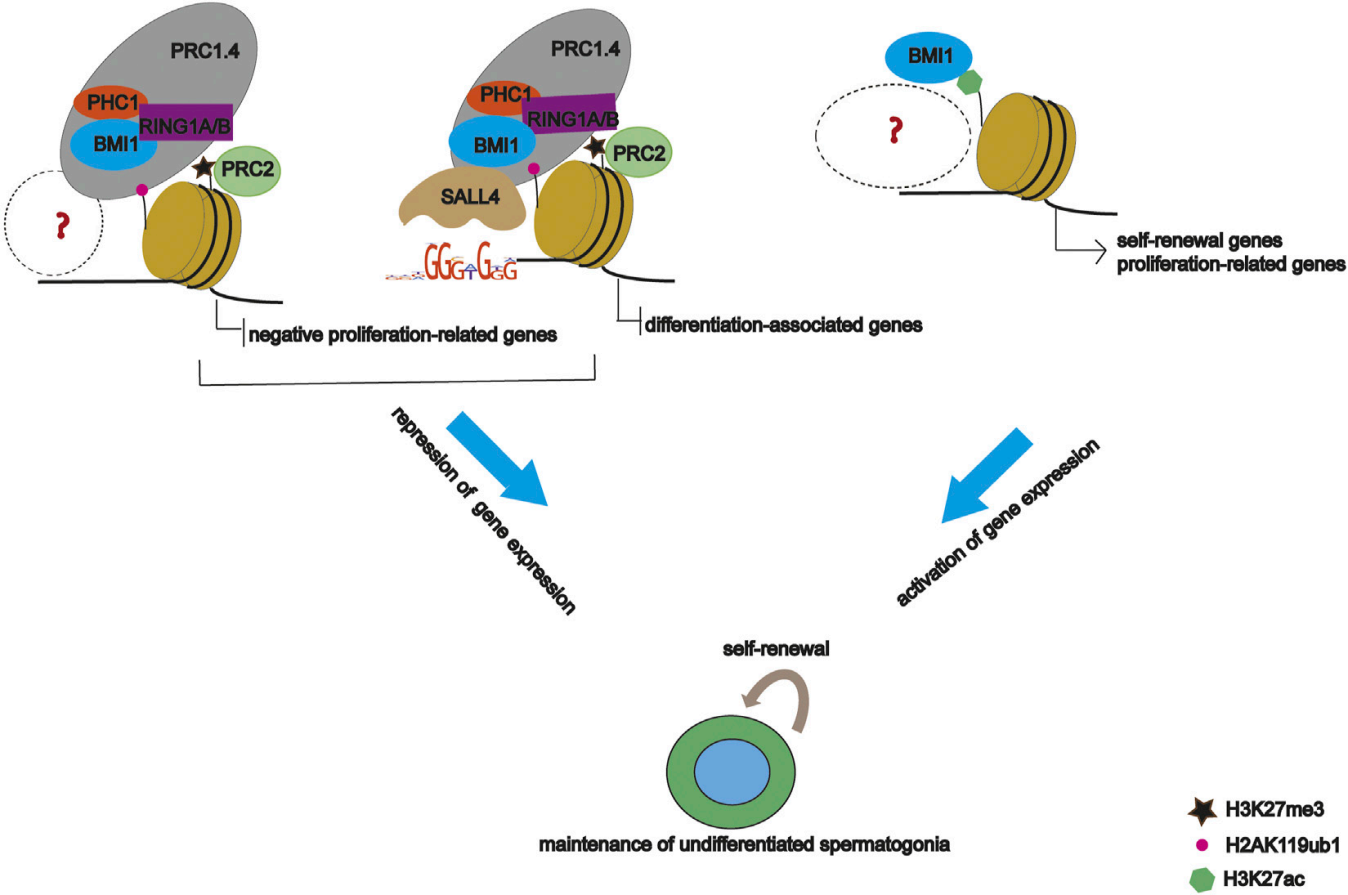
Proposed model of BMI1 regulation in undifferentiated spermatogonia.

Based on our data, we propose a model whereby BMI1 maintains undifferentiated spermatogonia by regulating gene expression through multiple distinct mechanisms (Figure 7). Specifically, BMI1 plays dual role in gene expression, and represses gene expression via interactions with other DNA binding proteins such as SALL4. However, given that PRC1 recruitment to genome is dependent on co-factors, further study is required to identify other co-factors mediating BMI1 recruitment.

In brief, this study characterizes the binding repertoire for BMI1 in undifferentiated spermatogonia. These findings provide evidence to understand the mechanisms by which BMI1 regulates target genes and the biological consequences of such regulation that are required for maintenance of undifferentiated spermatogonia. Our data will also serve as a resource to the scientific community to explore the molecular mechanisms involved in germ cell fate decisions in spermatogenesis.

## Data Availability

The datasets presented in this study can be found in online repositories. The names of the repository/repositories and accession number(s) can be found below: https://www.ebi.ac.uk/metagenomics/, E-MTAB-11777; https://www.ebi.ac.uk/metagenomics/, E-MTAB-11778; https://www.ncbi.nlm.nih.gov/geo/, GSE73390.
