# Supplementary figures and images for: BMI1 fine-tunes gene repression and activation to safeguard undifferentiated spermatogonia fate
Source: Front Cell Dev Biol. 2023 Apr 24;11:1146849. doi: 10.3389/fcell.2023.1146849 (PMC10164956; doi:10.3389/fcell.2023.1146849)

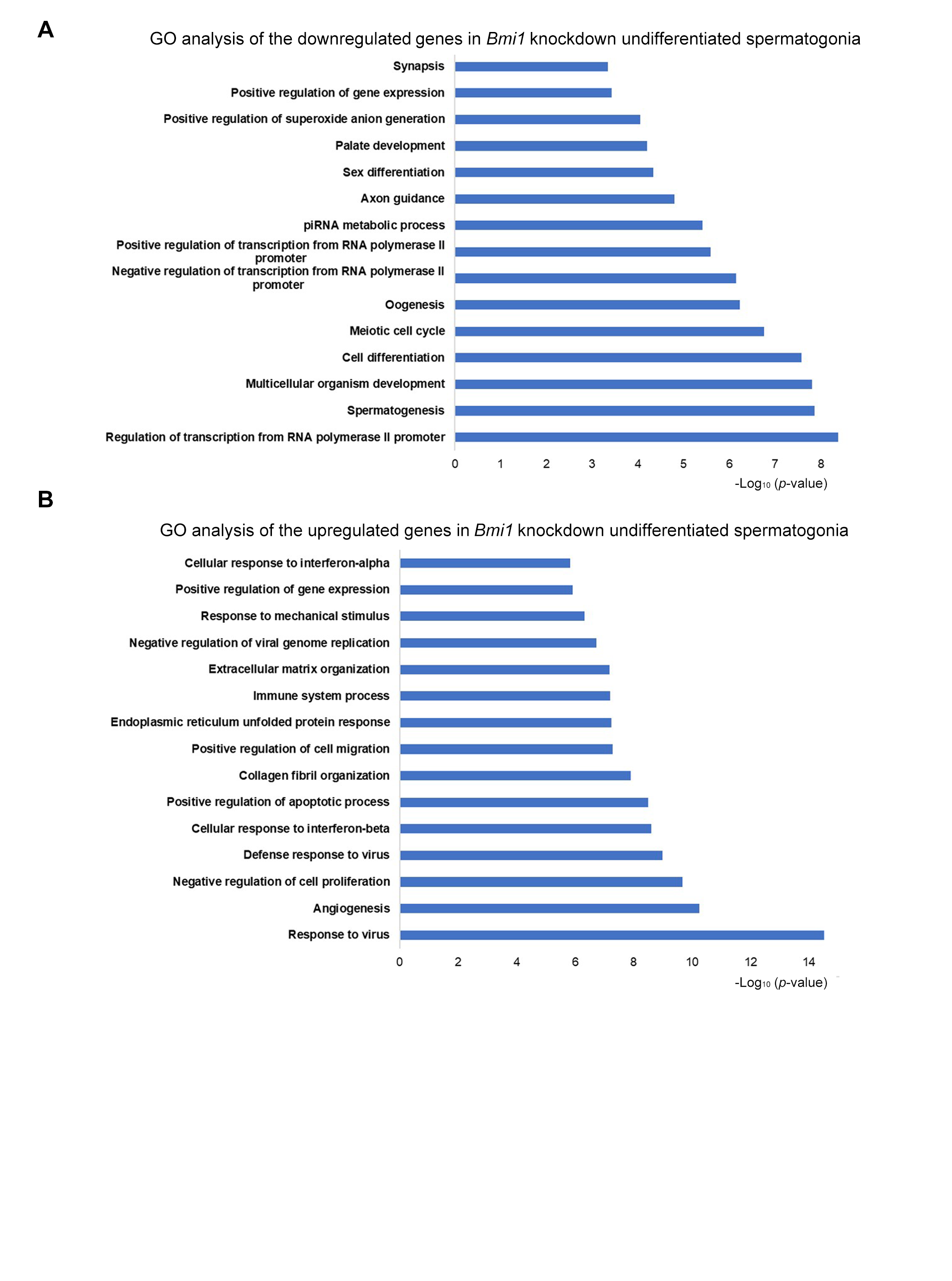

Supplement: Supplementary file 3 [file Image3.TIF]

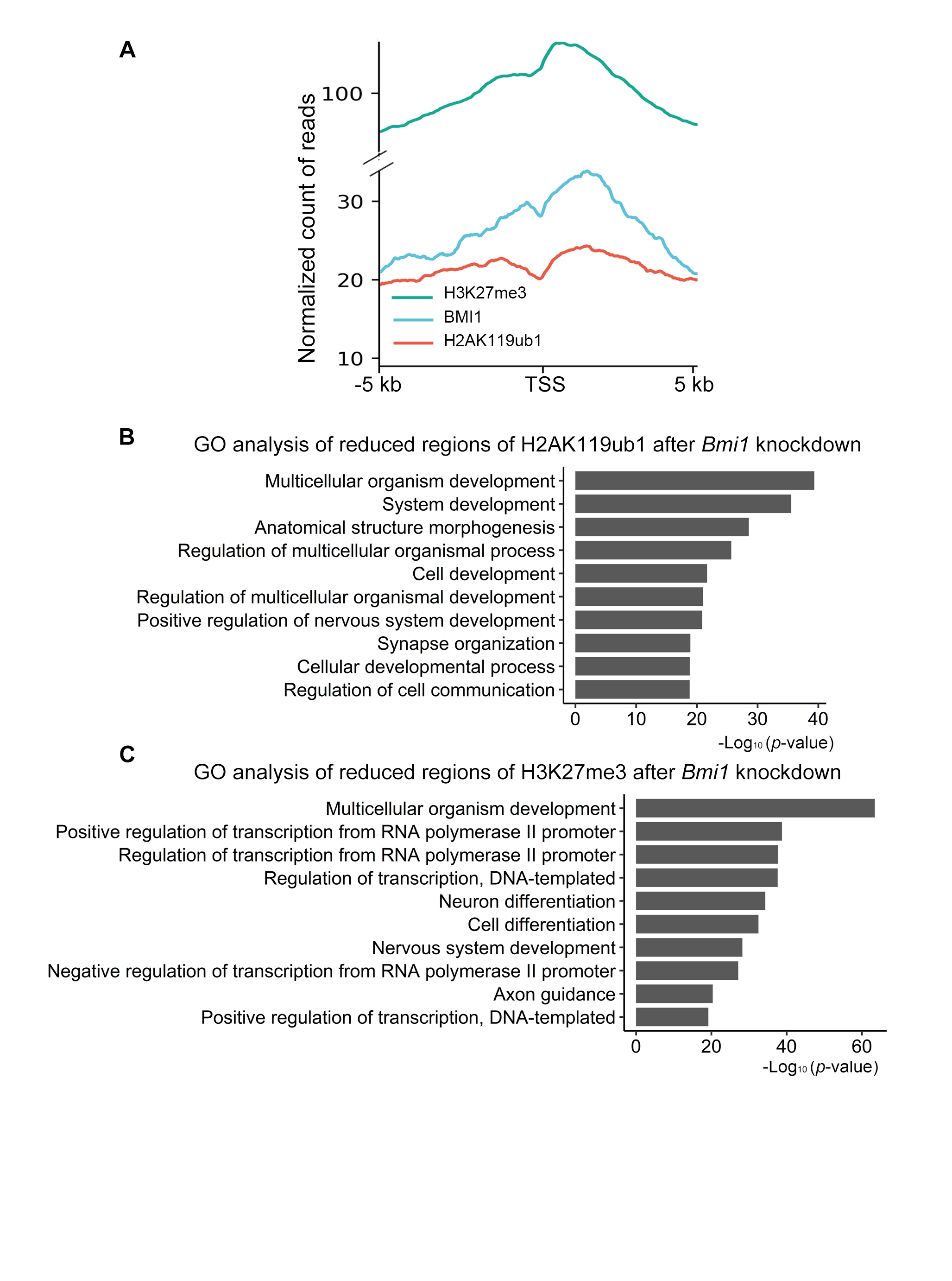

Supplement: Supplementary file 4 [file Image4.TIF]

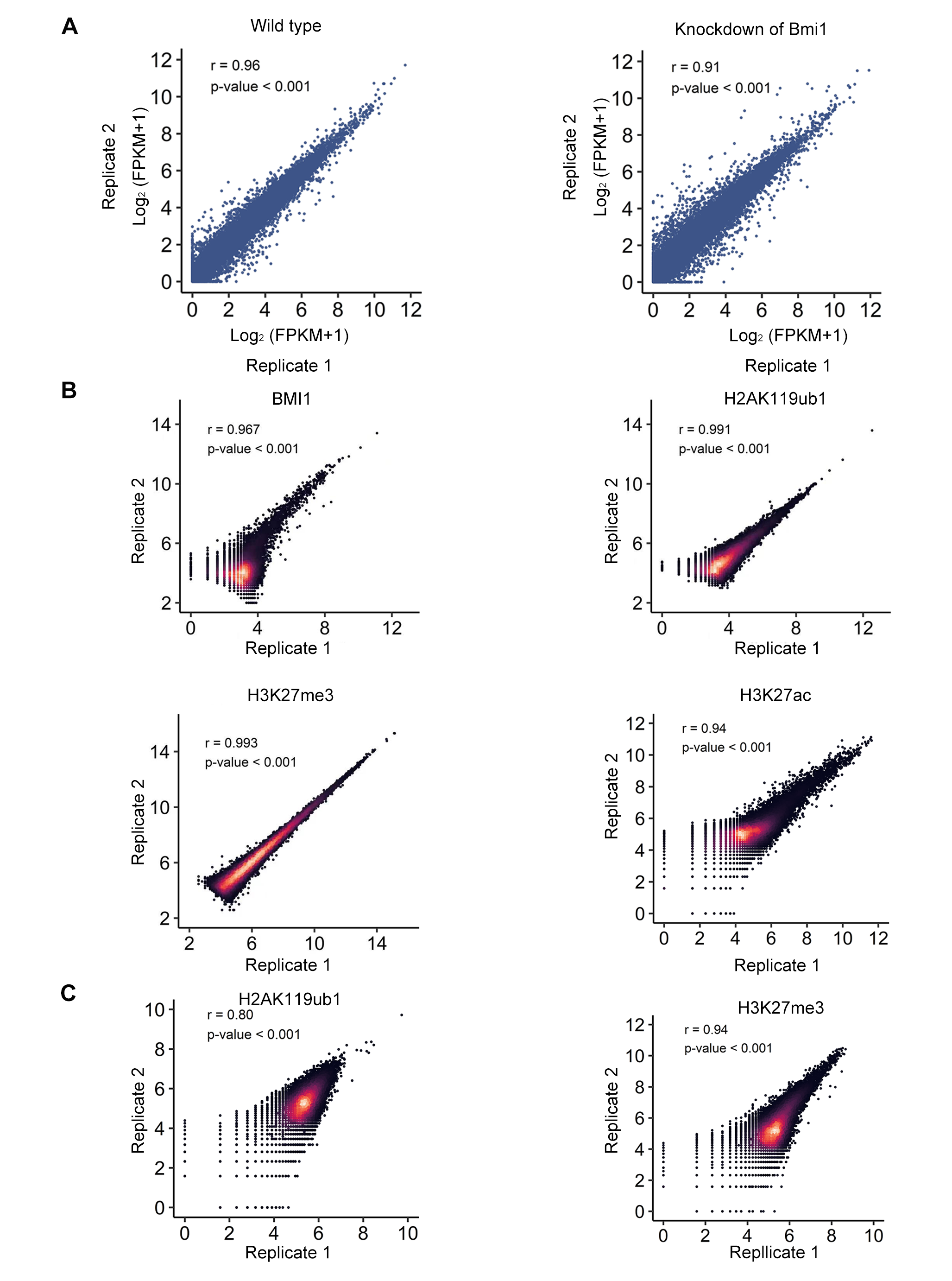

Supplement: Supplementary file 5 [file Image2.TIF]

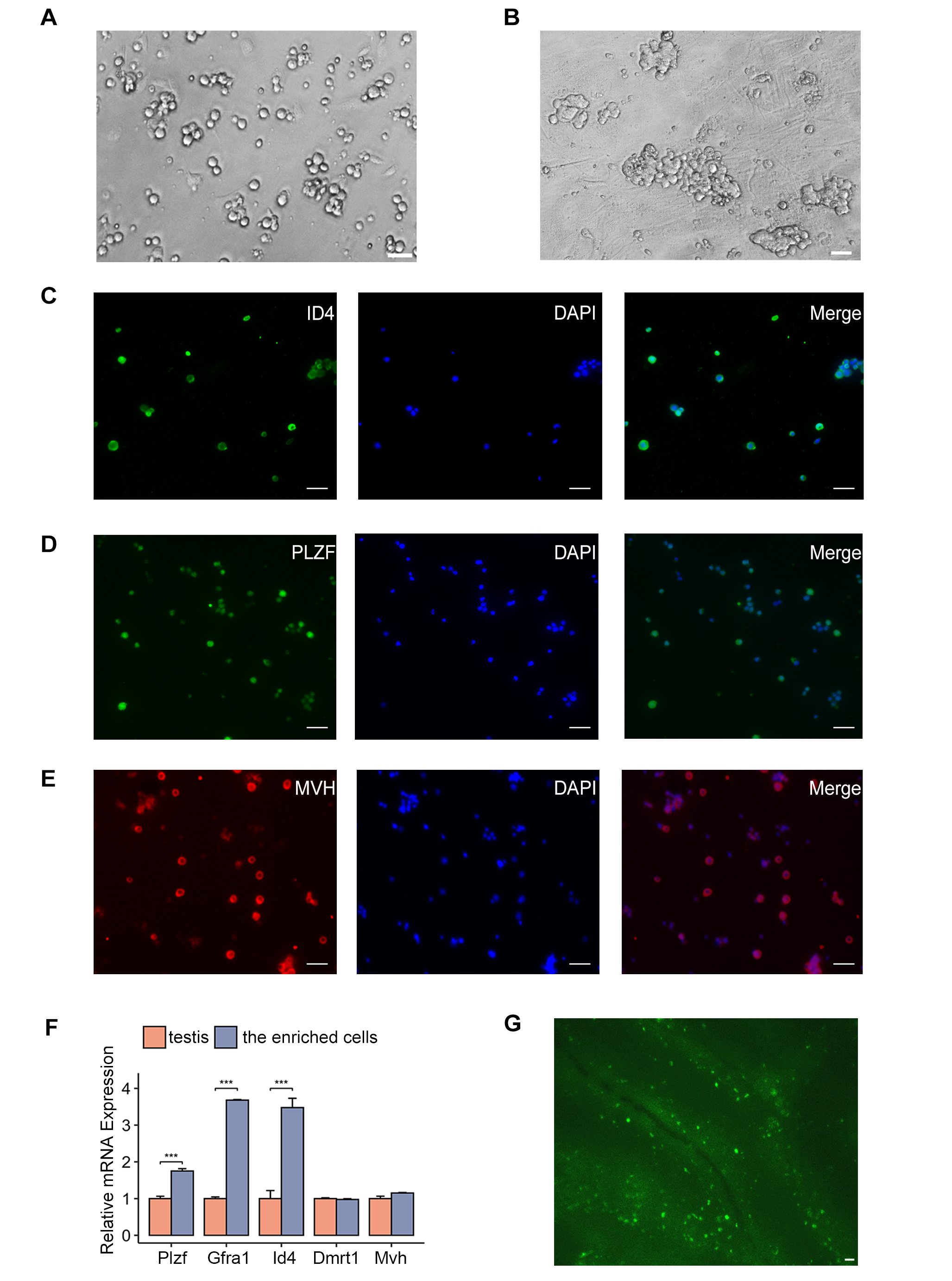

Supplement: Supplementary file 7 [file Image1.TIF]
