# Supplementary material for: BMI1 fine-tunes gene repression and activation to safeguard undifferentiated spermatogonia fate
Source: Front Cell Dev Biol. 2023 Apr 24;11:1146849. doi: 10.3389/fcell.2023.1146849 (PMC10164956; doi:10.3389/fcell.2023.1146849)
Supplement: Supplementary file 6 [file Table7.DOCX]

**Supplemental information**

**Supplemental Figures**

**Figure S1.** Identification of undifferentiated spermatogonia. **(A)** Morphology of the newly enriched undifferentiated spermatogonia on MEFs. Scale bar, 20 μm. (**B)** Grape-like string morphology of enriched undifferentiated spermatogonia after cultured 3 days upon MEFs. Scale bar, 20 μm. (**C)** IF of ID4 (green) in enriched undifferentiated spermatogonia. (**D)** IF of PLZF (green) in enriched undifferentiated spermatogonia. (**E)** IF of MVH (red) in enriched undifferentiated spermatogonia. Nuclei (c-e) are stained by DAPI (blue). Scale bar (c-e), 20 μm. (**F)** RT-qPCR analysis of undifferentiated spermatogonia and germline marker genes. Data are showed as means ± s.d. and derived from three independent experiments. (**G)** Image of the recipient testicular tubule after GFP-labelled undifferentiated spermatogonia transplantation

**Figure S2.** The reproducibility analysis of high-throughput sequencing data. (**A)** Correlation analysis of RNA-seq replicate data (including wild and *Bmi1* knockdown sample). (**B)** Correlation analysis of ChIP-seq replicate data in wild sample (including BMI1, H2AK119ub1, H3K27me3 and H3K27ac). (**C)** Correlation analysis of ChIP-seq replicate data in *Bmi1* knockdown sample (including H2AK119ub1 and H3K27me3)

**Figure S3.** Characterization of the BMI1-induced gene expression in undifferentiated spermatogonia. (**A)** GO analysis of downregulated genes in *Bmi1* knockdown cells. Results are expressed as -log_10_ (*p*-value). (**B)** GO analysis of upregulated genes in *Bmi1* knockdown cells. Results are expressed as -log_10_ (*p*-value).

**Figure S4.** Effect of *Bmi1* knockdown on the distribution of H2AK119ub1 and H3K27me3. (**A)** TSS (±5 kb) enrichment plot of BMI1, H2AK119ub1 and H3K27me3 ChIP-seq at target sites. (**B)** GO analysis of reduced regions of H2AK119ub1 after *Bmi1* knockdown. (**C)** GO analysis of reduced regions of H3K27me3 after *Bmi1* knockdown
